# Comorbidities of primary headache disorders: a literature review with meta-analysis

**DOI:** 10.1186/s10194-021-01281-z

**Published:** 2021-07-14

**Authors:** Valeria Caponnetto, Manuela Deodato, Micaela Robotti, Maria Koutsokera, Valeria Pozzilli, Cristina Galati, Giovanna Nocera, Eleonora De Matteis, Gioacchino De Vanna, Emanuela Fellini, Gleni Halili, Daniele Martinelli, Gabriele Nalli, Serena Serratore, Irene Tramacere, Paolo Martelletti, Alberto Raggi, On behalf of the European Headache Federation School of Advanced Studies (EHF-SAS)

**Affiliations:** 1 SS Filippo & Nicola Hospital , University of L’Aquila, Avezzano, Italy; 2 Department of Life Sciences, University of Trieste, Trieste, Italy; 3 Department of Medical, Surgical and Health Sciences, University of Trieste, Trieste, Italy; 4 Centro di Diagnosi e Cura delle Cefalee, Palazzo della Salute, Gruppo San Donato, Milano, Italy; 5 PainClinicMilano, Centro Medico Visconti di Modrone, Milano, Italy; 6 Thriassio General Hospital of Elefsina, Elefsina, Greece; 7 Internal Medicine Unit, Department of Clinical and Molecular Medicine, Sapienza University, Rome, Italy; 8 UO Neuropsichiatria Infantile, Policlinico Universitario Paolo Giaccone, Università degli Studi di Palermo, Palermo, Italy; 9 Neuroscience Section, Department of Applied Clinical Sciences and Biotechnology, University of L’Aquila, L’Aquila, Italy; 10 Clinica Neurologica, Dipartimento di Medicina, Università degli Studi di Perugia, Perugia, Italy; 11 Department of Neurology, University Hospital Center ‘Mother Teresa’, Tirana, Albania; 12 Department of Brain and Behavioral Sciences, University of Pavia, Pavia, Italy; 13 Headache Science and Neurorehabilitation Center, IRCCS Mondino Foundation, Pavia, Italy; 14 Dipartimento di Ricerca e Sviluppo Clinico, Fondazione IRCCS Istituto Neurologico Carlo Besta, Milano, Italy; 15 Department of Clinical and Molecular Medicine, Sapienza University, Roma, Italy; 16 Regional Referral Headache Center, Sant’Andrea University Hospital, Roma, Italy; 17 UOC Neurologia, Salute Pubblica, Disabilità, Fondazione IRCCS Istituto Neurologico Carlo Besta, Milano, Italy

**Keywords:** Primary headache, Migraine, TTH, CH, Comorbidity, Depression, Obesity, Diabetes, Hypertension, Stroke, Sleep disorders

## Abstract

**Background:**

Primary headache disorders are common and burdensome conditions. They are associated to several comorbidities, such as cardiovascular or psychiatric ones, which, in turn, contribute to the global burden of headache. The aim of this study is to provide a comprehensive description of the pooled prevalence of comorbidities of primary headache disorders using a meta-analytical approach based on studies published between 2000 and 2020.

**Methods:**

Scopus was searched for primary research (clinical and population studies) in which medical comorbidities were described in adults with primary headache disorders. Comorbidities were extracted using a taxonomy derived from the Global Burden of Disease (GBD) study. We compared prevalence of comorbidities among headache sufferers against general population using GBD-2019 estimates, and compared comorbidities’ proportions in clinical vs. population studies, and by age and gender.

**Results:**

A total of 139 studies reporting information on 4.19 million subjects with primary headaches were included: in total 2.75 million comorbidities were reported (median per subject 0.64, interquartile range 0.32–1.07). The most frequently addressed comorbidities were: depressive disorders, addressed in 51 studies (pooled proportion 23 %, 95 % CI 20–26 %); hypertension, addressed in 48 studies (pooled proportion 24 %, 95 % CI 22–26 %); anxiety disorders addressed in 40 studies (pooled proportion 25 %, 95 % CI 22–28 %). For conditions such as anxiety, depression and back pain, prevalence among headache sufferers was higher than in GBD-2109 estimates. Associations with average age and female prevalence within studies showed that hypertension was more frequent in studies with higher age and less females, whereas fibromyalgia, restless leg syndrome, and depressive disorders were more frequent in studies with younger age and more female.

**Conclusions:**

Some of the most relevant comorbidities of primary headache disorders – back pain, anxiety and depression, diabetes, ischemic heart disease and stroke – are among the most burdensome conditions, together with headache themselves, according to the GBD study. A joint treatment of headaches and of these comorbidities may positively impact on headache sufferers’ health status and contribute to reduce the impact of a group of highly burdensome diseases.

**Supplementary Information:**

The online version contains supplementary material available at 10.1186/s10194-021-01281-z.

## Introduction

The Global Burden of Diseases, Injuries, and Risk Factors Study (GBD) 2019 showed that headache disorders ranked third out of 369 conditions in terms of years lived with disability (YLDs) for both sexes, and the first in people aged 15–49 (accounting for 8 % of all-cause YLDs), with migraine ranking second and accounting for 7.3 % of all-cause YLDs [1, 2]. However, GBD estimates are biased by the adjustment for comorbidities, which is based on the assumption of the independent distribution of comorbid conditions. This is particularly critical as headache disorders have comorbidity for a variety of conditions, and headache – at least as a symptom – is a common experience to anyone, with any kind of health condition. The presence of multiple medical conditions, which constitutes part of the multifaceted and fragmented burden of headache disorders, is likely to lead to an increase of headache-related disability and cost for societies [3, 4].

Extensive research has recognized an association between primary headache and various comorbidities, as shown in some literature reviews [3, 5–10]. Comorbidities of primary headache disorders, include neurological, metabolic and cardiovascular conditions, e.g. stroke, epilepsy, multiple sclerosis, obesity, diabetes, hypertension, sleep disorders. In addition to these, mental health conditions, such as depression or anxiety, have been outlined: however, these comorbidities are sometimes poorly defined and addressed as symptoms of depression or anxiety. The same applies to chronic pain disorders for example fibromyalgia, low back pain or neck pain, and other musculoskeletal disorders [3, 6–8].

This constellation of comorbidities complicates the clinical management and the outcomes of primary headache, especially in chronic forms, where symptoms overlap [6, 7]. It is still difficult to determine through what mechanisms the conditions become comorbid. Comorbidity may act as risk factor for chronicity or as trigger for headache. Comorbidity may be a consequence of repeated headache attacks or headache treatments or a sequelae of other factors shared with headache [3, 5–7], and comorbidities are among the main drivers of chronification in migraine sufferers [11, 12]. Most of the available research on comorbidities of primary headaches is focused on migraine, with little appraisal of the comorbidities of tension-type headache (TTH) and cluster headache (CH).

Therefore, understanding the bidirectional relationships between primary headaches and presence of specific comorbidities may provide epidemiological and clinical clues concerning the pathophysiological mechanisms, the progression from episodic to chronic form, the appropriate diagnosis and treatments. In addition, a better knowledge of comorbidity in primary headache could contribute to drive the therapeutic symptomatic and prophylactic approaches, both pharmacological and non-pharmacological [13]. Indeed, non-pharmacological approaches, such as nutraceuticals, non-invasive neurostimulation, behavioral therapies and physical therapy, represent valid complementary options especially for patients with specific comorbidities, for those overusing medication, or for pregnant women [14–17]. A major awareness of the role of comorbidity in primary headache may therefore help clinicians in clinical management, improve headache sufferers’ quality of life and reduce impact on societies, defined in terms of disability, cost or reduced work productivity [4, 18–22].

Currently, there are no pooled data on comorbidity in primary headache, as research has mostly investigated specific relations, and therefore provided bidirectional information on relations such as migraine-hypertension, TTH-musculoskeletal disorders or CH-bipolar disorder. However, the simultaneous presence of primary headaches disorder and multiple medical conditions has not been subject to a full meta-analytic approach. Therefore, we currently have a partial understanding of the comorbidities that clinicians working with patients suffering from headache disorders may find in daily clinical practice.

The aim of present study is to provide a comprehensive description of the main comorbidities of primary headaches, i.e. migraine, TTH and CH, using a meta-analytical approach based on clinical studies and population surveys carried out between 2000 and 2020.

## Methods

We conducted a literature review with meta-analysis and reported results according to the ‘Preferred Reporting Items for Systematic Reviews and Meta-Analyses’ (PRISMA) [23].

### Search strategy

In order to identify suitable keywords for the search strategy, a pilot search was performed in Scopus and PubMed Mesh terms. All detected synonyms of ‘migraine’, ‘tension type headache’, ‘cluster headache’, ‘headache’ and ‘medication overuse headache’ (21 terms in total) were combined with the keywords ‘comorb*’, ‘multimorb*’, and all the keywords described as comorbidities of primary headaches, as described in the literature retrieved through a pilot search (66 terms in total). A search on Scopus covering the period between January 1st 2000 and October 9th, 2020 for primary research papers published in English and with an abstract was performed. Review keywords were searched in titles and abstracts: retrieved results were filtered according to relevant subject area (e.g. material sciences, arts and humanities, veterinary, energy) to exclude studies reporting non pertinent keywords. Extended search string is described in Table 1 of the Supplementary file. Retrieved references were managed with Endnote Web.

### Study selection

Retrieved references were equally and randomly assigned to twelve authors who screened titles and abstracts for eligibility. Three authors (VC, MD and VP) performed the double check about titles and abstracts eligibility of 20 % randomly selected references. To be eligible and be evaluated in full texts, titles and abstracts had to focus on primary headache disorders in adults: case reports letters, commentaries, editorials, reviews, and congress proceedings were excluded. In this phase, the agreement among the judgements of the raters (inter-rater reliability) was estimated with Krippendorff’s alpha coefficient (α) ranging from 0 (totally disagree) to 1 (totally agree). Any disagreement was resolved by discussion with a third author (AR) until consensus was reached.

Eligible references were equally and randomly assigned to fourteen authors who screened full texts for inclusion. For full texts evaluation, studies had to: (a) be available in full text; (b) be published on peer-reviewed journals in English; (c) include primary research (i.e. case reports letters, commentaries, editorials, reviews, and congress proceedings were excluded); (d) include adult subjects; (e) include subjects with primary headache disorders only, or studies with both primary and secondary headache disorders if the different group of subjects could be addressed separately for frequency of comorbidities (i.e. we included studies on both primary and secondary headache disorders if comorbidities could be referred to the subjects with primary headaches, by “downsizing” the sample accounting only for those with primary headaches). Studies reporting subjects with medication overuse headache were included only if they specified which was the underlying primary headache disorder, such as chronic migraine or chronic TTH, which was then extracted. All authors performed a double check on 50 % of the full texts and Krippendorff’s α was calculated: the choice for such a high rate is due to the large set of co-authors.

### Data extraction

Data extraction was performed through an ad hoc electronic spreadsheet of Microsoft Excel for Windows. Included studies were equally and randomly assigned to fourteen authors who extracted the following information: study type, i.e. clinical study vs. population survey; number of involved subjects for each type of primary headache, i.e. migraine, TTH, CH and trigeminal autonomic cephalalgias (TACs), and other primary headaches; when available, the total number of subjects and number of females, the average age (mean or median as available), the number of employed subjects, and the frequency of headache reported as monthly headaches were extracted too. If the information was not directly available (e.g. females referred as percentage, or headache frequency on a three-month basis), it was calculated.

For each study, the total number of comorbidities was extracted relying on the total number of subjects included in each study and not on the single primary headaches. The only exception was for those studies in which subjects with both primary and secondary headaches were included, and comorbidities referred to subjects with primary headache could be extracted separately from the other studies’ participants: in this cases, the total sample was “downsized” to that of participants of interest for our review. The choice of referring to the whole sample level (with the aforementioned exception) is due to the fact that in most of the studies comorbidities were reported at the whole sample level only, and not by showing the share of comorbidities by different primary headaches, e.g. by migraine vs. TTH, or by episodic vs. chronic migraine.

In order to extract comorbidities, the classification used by the recent publications of the GBD, which comprises a total of 105 non-communicable diseases, was used (see http://ghdx.healthdata.org/gbd-results-tool). Such a taxonomy included higher-level categories (e.g. Mental health disorders) and lower-level ones (e.g. Depressive disorders, Bipolar disorder, Psychotic disorders, Anxiety disorders) and a “Other” category in which those not included in the main categories are included: for example, “Other mental disorders” might include dissociative disorders or gender dysphoria. Once data were extracted, in case some comorbidities were reported by less than 2.5 % of the studies, then these were reclassified into the “other condition” by main disease type. Once the full set of comorbidities from the pre-defined list was completed, the “other” categories were revised in order to identify possible recurrent conditions that were not included in the GBD-derived list. If a condition was included in more than 2.5 % of the studies, then it was addressed as a stand-alone comorbidity.

### Data analysis

We descriptively summarized data reported to provide an overview of the included studies and samples in the studies, using medians and interquartile ranges (IQR) for raw data.

The measure of interest was the proportion of subjects with each single comorbidity among subjects with primary headache. The 95 % Confidence Intervals (95 %CI) were based on Wilson’s procedure [24]. The meta-analytic estimates were derived using random-effects models [25], and the pooled estimates were calculated after Freeman-Tukey Double Arcsine Transformation to stabilize variance [26]. The heterogeneity among studies was assessed relying on the χ^2^-test [27], and significant heterogeneity was defined when P-value was below 0.10. Inconsistency was quantified using the I^2^ statistic [28]: I^2^ below 40 % indicates no or not relevant heterogeneity; I^2^ comprised between 30 and 60 % indicates moderate heterogeneity: I^2^ comprised between 50 and 90 % indicates substantial heterogeneity; I^2^ higher than 75 % indicates considerable heterogeneity [29].

To address whether the pooled comorbidity rates observed among subjects with primary headaches are different from those reported in the general population, we relied on GBD-2019 estimates (available at: http://ghdx.healthdata.org/gbd-results-tool). Estimates herein presented are referred to prevalence ad based on all-age percentages, for both genders and at the global level. We searched for conditions that corresponded to those we extracted here but did not include the residual category “other” as the comparison is performed on different set of conditions: “other conditions” in our set is likely to include some unique causes in GBD list (e.g. aortic aneurysm was moved to “other cardiovascular disease”), and vice versa (e.g. restless leg syndrome was a unique comorbidity in our list but not in GBD one). We considered that prevalence of comorbidities is different among headache sufferers than in the general population if the 95 %CI of the pooled rates derived from our meta-analysis does not overlap with the 95 % Uncertainty Interval (95 %UI) of GBD-2019 estimates. The analytical approach herein described is at first reported considering all studies together.

A set of subgroup analyses was then carried out, namely: by study type, gender and age. Comparison by study type was performed by comparing clinical and population studies whereas for the latter subgroup analysis, the included studies were divided into two groups based on the corresponding median value observed for the percentage of females included in the studies and for the average age of participants. Therefore, we compared studies with a proportion of females ≥ than the median value calculated on all studies against those with proportion of females < than the median value, and studies with average age ≥ than the median average age reported in all studies against those with average age < than the median. For these sub-analyses, we did not include the residual category “other conditions” as the content is variable paper by paper and therefore the comparison by study type, average age and female prevalence is likely performed on different conditions.

## Results

**Fig. 1:**
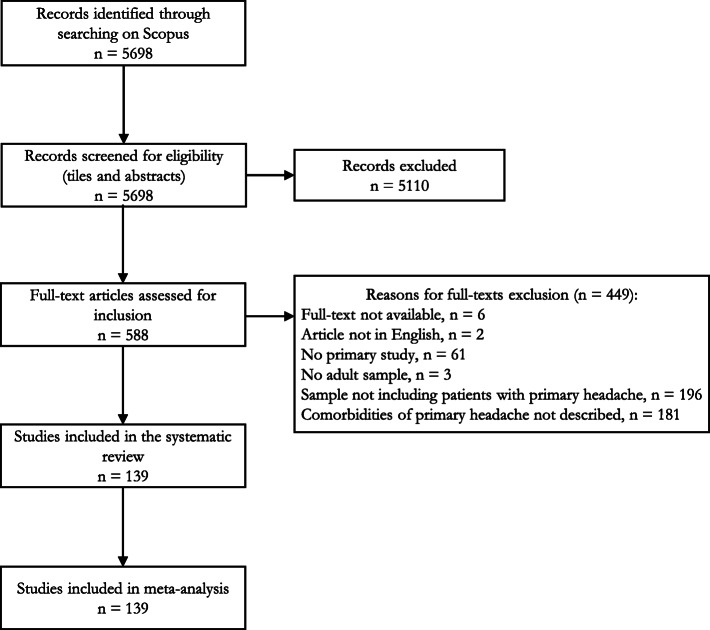
Flowchart of studies' selection

The electronic searches in Scopus identified 5698 potentially relevant records. Available full-texts of 588 records were then analyzed and we included 139 studies for the meta-analysis of results [30–168]. The PRISMA flow-chart is reported in Fig. 1. At abstract check, Krippendorff’s α was 0.90, at full-text it was 0.91.

Table 1 shows a synthesis of studies’ main features. The total number of respondents to the studies herein included was 4.19 million, with three studies accounting for the vast majority of persons (3.7 millions): most of the studies, 100 out of 139, were on participants with migraine only. Information on average age was reported in 131 studies: median average age was 40.4, IQR 36.9–46.0. Information on the amount of women per study was reported in 134 studies: median percentage of females was 77.8 %, IQR 71.4–90.0 %.

**Table 1:** Overview of selected studies

Main headache disorder by study	No. Studies	No. Patients	% of Females Median (IQR)	Average age Median (IQR)	Average monthly headaches Median (IQR)
Migraine only ^a^	100	3,455,438	79.7 % (72.6–91.5 %)	39.6 (36.4–46.0)	7.1 (4.9–9.8)
TTH only ^b^	5	14,100	53.0 % (53.0-60.5 %)	42.7 (42.7–42.8)	1.9 (1.5-2.0)
CH only ^c^	5	594	25.8 % (18.2–62.2 %)	49.1 (46.1–53.0)	NA
Mixed Studies^d^	29	724,072	77.4 % (72.5–89.2 %)	39.9 (37.9–42.9)	9.7 (4.6–13.8)
All studies	139	4,185,906	77.8 % (71.4–90.0 %)	40.4 (36.9–46.0)	7.0 (4.0-10.1)

Note. ^a^Among patients included in studies on migraine only, 48,650 (1.4 %) had episodic migraine, 4,651 (0.1 %) had chronic migraine, and 3,402,137 (98.5 %) had migraine not otherwise specified. In total, 13,765 (0.4 %) were enrolled in clinical studies and 3,441,673 (99.6 %) were enrolled in population studies

^b^Among patients included in studies on TTH only, 1,221 (8.7 %) had episodic TTH, 570 (4.0 %) had chronic TTH, and 12,309 (87.9 %) had TTH not otherwise specified. In total, 81 (0.6 %) were enrolled in clinical studies and 14,019 (99.4 %) were enrolled in population studies

^c^Among patients included in studies on CH only, 455 (76.6 %) had episodic CH, and 133 (22.4 %) had chronic CH, 6 (1.0 %) had other TACs. All patients were enrolled in clinical studies

^d^Among patients included in studies on mixed headache disorders, 137,118 (18.9 %) had migraine, of whom 132,167 had episodic migraine; 568,986 (78.6 %) had TTH, of whom 568,110 had episodic TTH; 24 (< 0.1 %) had CH or other TACs, of whom 13 had episodic CH; 18,044 (24.9 %) had other primary headache, of whom 339 were patients with both migraine-like and TTH-like headaches and 1667 had chronic daily headache not better specified, 2,337 had other primary headache not better specified and 12,571 had other non-migraine primary headache

A total of 33 main comorbidities, including five large-group categories referred as “any” (e.g. Any cancer, Any substance use disorder) and seven additional groups referred as “others” (e.g. Other cancers, Other neurological disorders), were reported in more than 2.5 % of the studies. In total, among the aforementioned 4.19 million subjects, 2.75 million comorbidities were reported, with a median ratio of comorbidities per subject of 0.64 (IQR: 0.32–1.07), with 39 studies reporting a comorbidity ratio per subject higher than 1.0. Table 2 of the Supplementary file shows the whole raw distribution of comorbidities across the different studies.

Table 2 shows a synthesis of the pooled distribution of the main comorbidities across all studies, as well as the contrast to GBD 2019 estimates. The most frequently addressed comorbidities were: depressive disorders, hypertension, anxiety disorders and diabetes, addressed in 51, 48, 40 and 39 studies, respectively. Excluding the conditions referred as “other”, the comorbidities with the highest pooled proportion were oral disorders (67 %, 95 %CI 40–89 %; reported in nine studies); sleep disorders (48 %, 95 %CI 42–54 %; reported in 30 studies); back pain (46 %, 95 %CI 20–72 %; reported in seven studies); anxiety disorders (25 %, 95 %CI 22–28 %; reported in 40 studies). See Supplementary Figures for the forest plot of each comorbidity.

**Table 2:** Pooled prevalence of selected comorbidities among patients with primary headaches and comparison with 2019 GBD Estimates

Associated condition category	Associated condition	No. of studies	Pooled proportion (95 % CI)	GBD Estimates Mean (95 % UI)	Comparison
Cancers	Any cancer	4	5 % (0–13 %)	6.5 % (5.6–7.6 %)	
Cardiovascular diseases	Ischemic heart disease	19	7 % (5–10 %)	2.6 % (2.4–2.9 %)	Higher in headaches
	Stroke/Cerebrovascular	30	5 % (3–7 %)	1.4 % (1.2–1.5 %)	Higher in headaches
	Hypertension	48	24 % (22–26 %)	Risk factor in GBD	
	Atrial fibrillation and flutter	6	2 % (1–3 %)	0.80 % (0.61–1.01 %)	
	Other cardiovascular and circulatory diseases	23	9 % (7–11 %)	–	
Digestive diseases	Upper digestive system diseases	4	10 % (4–19 %)	10.5 % (9.4–11.6 %)	
	Irritable bowel syndrome	4	19 % (1–53 %)	0.07 % (0.06–0.07 %)	Higher in headaches
	Other digestive diseases	7	14 % (1–37 %)	–	
Metabolic and kidney diseases	Diabetes mellitus	39	6 % (5–7 %)	6.2 % (5.7–6.7 %)	
	Chronic kidney disease	5	3 % (0–10 %)	9.4 % (8.7–10.0 %)	
	Obesity	26	21 % (17–26 %)	Risk factor in GBD	
	Hyperlipidemia	14	15 % (8–24 %)	Not in GBD	
	Hypercholesterolemia	8	26 % (12–43 %)	Risk factor in GBD	
	Thyroid diseases	9	12 % (8–16 %)	Not in GBD	
	Other Metabolic/Kidney disease	11	15 % (10–22 %)	–	
Musculoskeletal disorders	Arthritis	8	12 % (9–16 %)	7.1 % (6.4–7.9 %)	Higher in headaches
	Back pain	7	46 % (20–72 %)	7.6 % (6.8–8.6 %)	Higher in headaches
	Fibromyalgia	9	26 % (8–50 %)	Not in GBD	
	Other musculoskeletal disorders	4	27 % (5–52 %)	–	
Neurological disorders	Parkinson disease	5	1 % (1–2 %)	0.11 % (0.10–0.13 %)	Higher in headaches
	Idiopathic epilepsy	7	2 % (1–2 %)	0.34 % (0.26–0.42 %)	Higher in headaches
	Sleep disorder	30	48 % (42–54 %)	Not in GBD	
	Restless leg syndrome	16	20 % (13–27 %)	Not in GBD	
	Other neurological disorders	19	15 % (10–20 %)	–	
Mental health disorders	Depressive disorders	51	23 % (20–26 %)	3.8 % (3.4–4.2 %)	Higher in headaches
	Bipolar disorder	6	5 % (0–12 %)	0.53 % (0.44–0.63 %)	
	Anxiety disorders	40	25 % (22–28 %)	4.1 % (3.4–4.8 %)	Higher in headaches
	Post-Traumatic Stress Disorder	7	15 % (6–28 %)	Not in GBD	
	Other mental disorders	17	25 % (15–36 %)	–	
Respiratory diseases	Asthma	8	8 % (3–13 %)	3.5 % (3.0-4.2 %)	
	Chronic obstructive pulmonary disease	5	6 % (0–17 %)	2.8 % (2.7-3.0 %)	
	Other chronic respiratory diseases	6	13 % (0–41 %)	–	
Sense organ disease	Any sense organ disease	6	26 % (10–47 %)	23.7 % (22.5–24.9 %)	
Skin and subcutaneous disease	Any skin and subcutaneous disease	4	5 % (3–7 %)	27.2 % (26.5–28.0 %)	Higher in GBD
Substance use disorders	Any substance use disorder	10	6 % (0–17 %)	2.2 % (1.9–2.4 %)	
Other NCDs	Oral disorders	9	67 % (40–89 %)	46.8 % (43.0-50.7 %)	
	Hemoglobinopathies and hemolytic anemias	4	1 % (1–2 %)	28.4 % (27.3–29.6 %)	Higher in GBD
	Other disorders (Congenital birth defects & Gynecological diseases)	4	28 % (5–60 %)	–	
	Allergies	8	22 % (12–34 %)	23.7 % (22.5–24.9 %)	

For 23 single comorbidities, a corresponding GBD-2019 estimate was available. As shown in Table 2, for nine of them the pooled proportion derived from the present review was higher than the GBD-produced estimates, and the opposite happened for two of them; in the remaining 12, the 95 % CI of the pooled proportion herein defined overlapped with and the 95 % UI produced by GBD-2019 estimates. Specifically, higher rates were found for ischemic heart disease, stroke/cerebrovascular conditions, irritable bowel syndrome, arthritis, back pain, Parkinson's disease, idiopathic epilepsy, depressive disorders, and anxiety disorders.

### Subgroup analyses

Table 3 reports the results of the comparison performed by study type. Higher comorbidity proportions were observed in clinical studies, specifically for thyroid diseases, fibromyalgia, sleep disorder, restless leg syndrome (RLS), depressive disorders, anxiety disorders, post-traumatic stress disorder. On the contrary, higher rates were observed in population studies for arthritis, skin disorders, and allergies. Taken as a whole, it can be concluded that higher comorbidity rates are observed among samples enrolled in clinical studies (median comorbidity per subject 0.70, IQR 0.40–1.09) than among samples from population studies (median 0.50, IQR 0.29–0.97).

**Table 3:** Pooled comorbidity proportion by study type

Associated condition category	Associated condition	Population studies (52 studies)	Clinical studies (87 studies)	Heterogeneity between Groups
Cancers	Any cancer	3 % (3–3 %)	2 % (0–5 %)	*p* = .131
Cardiovascular diseases	Ischemic heart disease	8 % (5–11 %)	5 % (2–10 %)	*p* = .293
	Stroke/Cerebrovascular	4 % (3–7 %)	6 % (3–9 %)	*p* = .241
	Hypertension	26 % (24–29 %)	19 % (14–26 %)	*p* = .061
	Atrial fibrillation and flutter	1 % (0–2 %)	2 % (0–9 %)	*p* = .479
Digestive diseases	Upper digestive system diseases	17 % (16–18 %)	8 % (2–18 %)	*p* = .086
	Irritable bowel syndrome	–	19 % (1–53 %)	–
Metabolic and kidney diseases	Diabetes mellitus	6 % (4–7 %)	7 % (3–12 %)	*p* = .281
	Chronic kidney disease	4 % (0–13 %)	0 % (0–1 %)	*p* = .097
	Obesity	18 % (13–24 %)	25 % (16–35 %)	*p* = .203
	Hyperlipidemia	17 % (8–29 %)	13 % (9–19 %)	*p* = .543
	Hypercholesterolemia	29 % (13–49 %)	15 % (9–22 %)	*p* = .137
	Thyroid diseases	3 % (3–3 %)	16 % (7–27 %)	*p* < .001
Musculoskeletal disorders	Arthritis	19 % (15–23 %)	6 % (0–15 %)	*p* = .028
	Back pain	54 % (53–55 %)	48 % (11–86 %)	*p* = .774
	Fibromyalgia	1 % (1–1 %)	31 % (20–44 %)	*p* < .001
Neurological disorders	Parkinson disease	1 % (1–2 %)	–	–
	Idiopathic epilepsy	2 % (1–2 %)	2 % (2–3 %)	*p* = .199
	Sleep disorder	33 % (25–41 %)	56 % (46–66 %)	*p* = .001
	Restless leg syndrome	6 % (2–13 %)	28 % (20–38 %)	*p* < .001
Mental health disorders	Depressive disorders	15 % (11–19 %)	31 % (24–38 %)	*p* = .000
	Bipolar disorder	5 % (0–17 %)	3 % (2–5 %)	*p* = .799
	Anxiety disorders	18 % (16–21 %)	30 % (23–39 %)	*p* = .003
	Post-traumatic stress disorder	2 % (2–2 %)	21 % (16–26 %)	*p* < .001
Respiratory diseases	Asthma	9 % (3–16 %)	7 % (0–27 %)	*p* = .839
	Chronic obstructive pulmonary disease	6 % (0–21 %)	3 % (2–4 %)	*p* = .489
Sense organ disease	Any sense organ diseases	–	26 % (10–47 %)	–
Skin and subcutaneous disease	Any skin and subcutaneous disease	7 % (7–7 %)	1 % (0–3 %)	*p* < .001
Substance use disorders	Any substance use disorder	5 % (0–18 %)	7 % (4–10 %)	*p* = .665
Other NCDs	Oral disorders	–	67 % (40–89 %)	–
	Hemoglobinopathies and hemolytic anemias	3 % (3–3 %)	0 % (0–2 %)	*p* = .300
	Other disorders (Congenital birth defects & Gynecological diseases)	15 % (10–21 %)	33 % (0–85 %)	*p* = .430
	Allergies	33 % (17–51 %)	12 % (8–17 %)	*p* = .012

Table 4 reports the results of the comparison performed by female percentage across studies. Higher comorbidity proportions were observed in studies with a higher percentage of females for fibromyalgia, RLS, depressive disorders, and anxiety disorders. On the contrary, higher rates were observed in studies with a lower percentage of females (i.e. with a higher male percentage) for hypertension, asthma, sense organ diseases, skin disorders, and allergies. Taken as a whole, it can be concluded that difference in females’ prevalence across sample has a limited effect on total comorbidity rates, as the median and interquartile ranges were largely overlapping (median 0.66, IQR 0.34–1.03 for studies with higher female percentage; median 0.61, IQR 0.30–1.05 for studies with lower female percentage).

**Table 4:** Pooled comorbidity proportion by female percentage within studies

Associated condition category	Associated condition	Female percentage ≥ 77.8 % (67 studies)	Female percentage < 77.8 % (67 studies)	Heterogeneity between Groups
Cancers	Any cancer	2 % (2–5 %)	2 % (2–3 %)	*p* = .077
Cardiovascular diseases	Ischemic heart disease	5 % (1–10 %)	8 % (5–12 %)	*p* = .261
	Stroke/Cerebrovascular	3 % (2–4 %)	5 % (2–10 %)	*p* = .447
	Hypertension	18 % (15–21 %)	29 % (24–34 %)	*p* < .001
	Atrial fibrillation and flutter	1 % (1–1 %)	2 % (0–6 %)	*p* = .193
Digestive diseases	Upper digestive system diseases	12 % (5–22 %)	6 % (5–8 %)	*p* = .111
	Irritable bowel syndrome	7 % (4–9 %)	11 % (7–18 %)	*p* = .090
Metabolic and kidney diseases	Diabetes mellitus	5 % (3–8 %)	7 % (5–9 %)	*p* = .650
	Chronic kidney disease	–	3 % (0–10 %)	–
	Obesity	21 % (18–24 %)	21 % (12–32 %)	*p* = .924
	Hyperlipidemia	12 % (7–19 %)	17 % (8–29 %)	*p* = .419
	Hypercholesterolemia	22 % (8–41 %)	28 % (12–48 %)	*p* = .659
	Thyroid diseases	13 % (2–31 %)	11 % (7–16 %)	*p* = .638
Musculoskeletal disorders	Arthritis	11 % (0–30 %)	15 % (10–21 %)	*p* = .772
	Back pain	39 % (4–83 %)	66 % (65–67 %)	*p* = .234
	Fibromyalgia	31 % (25–36 %)	2 % (2–2 %)	*p* < .001
Neurological disorders	Parkinson disease	–	1 % (1–2 %)	–
	Idiopathic epilepsy	1 % (1–1 %)	2 % (1–3 %)	*p* = .112
	Sleep disorder	48 % (27–69 %)	47 % (41–53 %)	*p* = .950
	Restless leg syndrome	35 % (24–48 %)	9 % (4–16 %)	*p* < .001
Mental health disorders	Depressive disorders	29 % (23–36 %)	19 % (16–21 %)	*p* = .001
	Bipolar disorder	1 % (1–1 %)	7 % (0–21 %)	*p* = .147
	Anxiety disorders	32 % (24–40 %)	20 % (22–28 %)	*p* = .004
	Post-traumatic stress disorder	17 % (3–40 %)	10 % (8–13 %)	*p* = .491
Respiratory diseases	Asthma	2 % (2–3 %)	8 % (4–12 %)	*p* = .002
	Chronic obstructive pulmonary disease	2 (2–3 %)	7 % (0–22 %)	*p* = .342
Sense organ disease	Any sense organ diseases	7 % (4–10 %)	51 % (37–64 %)	*p* < .001
Skin and subcutaneous disease	Any skin and subcutaneous disease	1 % (0–3 %)	7 % (7–7 %)	*p* < .001
Substance use disorders	Any substance use disorder	7 % (0–27 %)	4 % (2–5 %)	*p* = .622
Other NCDs	Oral disorders	66 % (17–100 %)	69 % (53–82 %)	*p* = .898
	Hemoglobinopathies and hemolytic anemias	0 % (0–2 %)	3 % (3–3 %)	*p* = .300
	Other disorders (Congenital birth defects & Gynecological diseases)	33 % (0–85 %)	15 % (10–21 %)	*p* = .430
	Allergies	12 % (6–20 %)	29 % (15–45 %)	*p* = .043

Table 5 reports the results of the comparison performed by average age across studies. Higher comorbidity proportions were observed in studies with older subjects for hypertension, irritable bowel syndrome, chronic kidney disease, hypercholesterolemia, and chronic obstructive pulmonary disease. On the contrary, higher rates were observed in studies with younger subjects for sleep disorder, RLS, depressive disorders, and other disorders (congenital birth defects & gynecological diseases). Taken as a whole, it can be concluded that difference in average has a limited effect on total comorbidity rates, as the median and interquartile ranges were largely overlapping (median 0.63, IQR 0.34–0.97 for studies with younger participants; median 0.65, IQR 0.32–1.05 for studies with older participants).

**Table 5:** Pooled comorbidity proportion by average age of participants

Associated condition category	Associated condition	Average age ≤ 40.3 (65 studies)	Average age ≥ 40.4 (66 studies)	Heterogeneity between groups
Cancers	Any cancer	2 % (2–3 %)	2 % (0–5 %)	*p* = .077
Cardiovascular diseases	Ischemic heart disease	6 % (3–9 %)	6 % (3–10 %)	*p* = .908
	Stroke/Cerebrovascular	3 % (2–5 %)	4 % (1–8 %)	*p* = .591
	Hypertension	13 % (9–17 %)	30 % (27–32 %)	*p* < .001
	Atrial fibrillation and flutter	2 % (1–3 %)	1 % (0–3 %)	*p* = .813
Digestive diseases	Upper digestive system diseases	14 % (6–24 %)	3 % (1–7 %)	*p* = .010
	Irritable bowel syndrome	6 % (4–8 %)	19 % (13–28 %)	*p* < .001
Metabolic and kidney diseases	Diabetes mellitus	5 % (4–7 %)	6 % (4–8 %)	*p* = .709
	Chronic kidney disease	0 % (0–0 %)	5 % (1–11 %)	*p* = .007
	Obesity	21 % (15–29 %)	19 % (14–26 %)	*p* = .681
	Hyperlipidemia	10 % (3–20 %)	15 % (9–21 %)	*p* = .359
	Hypercholesterolemia	9 % (2–22 %)	38 % (30–36 %)	*p* < .001
	Thyroid diseases	13 % (4–24 %)	12 % (2–26 %)	*p* = .937
Musculoskeletal disorders	Arthritis	14 % (12–15 %)	10 % (6–16 %)	*p* = .470
	Back pain	67 % (60–74 %)	41 % (5–84 %)	*p* = .259
	Fibromyalgia	29 % (20–38 %)	13 % (0–48 %)	*p* = .382
Neurological disorders	Parkinson disease	–	1 % (1–2 %)	–
	Idiopathic epilepsy	2 % (1–4 %)	2 % (1–3 %)	*p* = .852
	Sleep disorder	60 % (40–79 %)	34 % (28–39 %)	*p* = .013
	Restless leg syndrome	32 % (19–46 %)	11 % (6–19 %)	*p* = .005
Mental health disorders	Depressive disorders	31 % (22–41)	19 % (15–22 %)	*p* = .011
	Bipolar disorder	1 % (1–2 %)	6 % (0–18 %)	*p* = .132
	Anxiety disorders	25 % (18–34 %)	24 % (21–28 %)	*p* = .841
	Post-traumatic stress disorder	12 % (10–14 %)	14 % (1–36 %)	*p* = .809
Respiratory diseases	Asthma	4 % (2–7 %)	9 % (4–14 %)	p = .056
	Chronic obstructive pulmonary disease	2 % (1–3 %)	12 % (12–13 %)	*p* < .001
Sense organ disease	Any sense organ diseases	49 % (38–60 %)	29 % (5–62 %)	*p* = .266
Skin and subcutaneous disease	Any skin and subcutaneous disease	–	5 % (3–7 %)	–
Substance use disorders	Any substance use disorder	2 % (0–5 %)	8 (1–22 %)	*p* = .164
Other NCDs	Oral disorders	66 % (57–74 %)	71 % (0-100 %)	*p* = .931
	Hemoglobinopathies and hemolytic anemias	–	1 % (1–2 %)	–
	Other disorders (Congenital birth defects & Gynecological diseases)	58 % (48–68 %)	6 % (4–9 %)	*p* < .001
	Allergies	32 % (30–34 %)	19 % (7–34 %)	*p* = .085

## Discussion

The results of this literature review with meta-analysis show that out of 4.19 million headache sufferers, 3.70 million comorbidities were reported (median 0.64, interquartile range 0.32–1.07). For many conditions, prevalence among subjects with primary headache disorders were higher than what can be estimated in the general population, with some conditions – in particular, depression, anxiety and back pain – showing pooled prevalence higher than 20 % in the lower bound. In addition to them, there are other comorbidities with a considerably high prevalence among headache sufferers, such as hypertension, sleep disorders and oral disorders: however, for the first two, no GBD estimates were available, whereas for the third, the estimates generated by our review overlapped with those referred to the general population. Data derived from clinical studies included a higher prevalence in some conditions, and a globally higher raw rate of comorbidity per subject, likely owing to a higher precision in comorbidities identification. Minor differences were instead retrieved from the age and gender comparison, but some specific associations could be observed for some of the most relevant comorbidities. Hypertension was mostly associated to older age and lower females’ prevalence; fibromyalgia, restless leg syndrome, and depressive disorders were mostly associate to younger age and higher females’ prevalence.

Our findings show that the most frequent psychiatric comorbidities in subjects with primary headaches were anxiety and depression, followed by post-traumatic stress disorder: these comorbidities were found, respectively, in 25 % (95 %CI: 22–28 %), 23 % (95 %CI: 20–26 %) and 15 % (95 %CI: 6–28 %) of the subjects. Several studies in literature confirmed the coexistence of these conditions in subjects with headaches and particularly in those with migraine [169–171].

Understanding psychiatric comorbidities in subjects suffering from headache disorders, and migraine in particular, is important in reason of the bidirectional relationship between the two [10]: in fact, anxiety and depression can determine the onset of headache, but they can be a consequence of frequent headache attacks [171]. These considerations are essential in clinical practice: comorbidity to anxiety and depression seems to have limited influence on the use and overuse of medications, but subjects with these comorbidities perceived a lower treatment satisfaction and effectiveness [172]. Regarding prophylactic treatment for chronic migraine (CM), there is an important overlap between drugs that are prescribed for CM, anxiety and mood disorders: in fact, antidepressants such as amitriptyline and anxiolytics such as bromazepam are commonly used in CM prophylaxis. As migraine, anxiety and depression share common neurobiological pathophysiology (e.g. derangement in central monoaminergic systems and abnormalities in the metabolism of glutamate and gamma-aminobutyric acid [173]), when present simultaneously should be treated with a single medication [174].

Screening for comorbid psychiatric disorders in headache sufferers, and among those with migraine in particular, is therefore of great importance for management, treatment and prognosis. Important alternative interventions that are worth adding to pharmacological treatment include non-pharmacological ones. In particular, behavioral therapies, such as cognitive behavioral therapy or mindfulness-based approaches, have shown to be useful in treating symptoms related to headache, but also anxiety and depression [15, 175, 176].

Prior studies have described a close correlation between headache and sleep disorders [177, 178], and such knowledge was enriched by our presentation of the pooled prevalence of sleep disorder (48 %, 95 %CI: 42–54 %) and RLS (20 %, 95 %CI: 13–27 %) in primary headaches. The relationships between primary headache and sleep disorders is poorly understood [178]: sleep disturbances may act as a trigger for headache, but headache may promote sleep disturbances which, in turn, may also be related to depressed mood. The comorbidity between headache disorders, anxiety and depression, and sleep-related disturbances is a driver for worse health outcomes [179]. These three comorbidities have a pivot role in pain modulation. Primary headaches are influenced by sleep wake cycle, with a probable involvement of the hypothalamus, which does not only regulate the sleep-wake cycle, but is also involved in pain modulation [177, 178, 180, 181]. In addition to this, a dysfunction of serotoninergic and dopaminergic pathways seems to explain the simultaneous presence of headache and sleep disorder: impairments of serotoninergic system are common among headache, sleep and psychiatric disorder, whereas impairments of dopaminergic system is common in headache, sleep disorders and RLS [177, 180, 181].

These findings have several implications for clinical practice: first, the evaluation of the sleep habits is of great relevance as they may be considered prognostic factors for primary headache development and a risk factor for shift to chronic headache; second, the combined multimodal approaches may be effective in improving headache parameters thought the joint treatment of sleep comorbidities.

There are conflicting results about the coexistence of migraine and diabetes. In our review, prevalence of diabetes among persons with headache was 6 % (95 %CI: 5–7 %), but few reports on such a relation exist. A recent study observed no significant differences in prevalence of migraine between patients with diabetes mellitus and healthy controls [182], whereas another study has shown that insulin resistance seems to exist in individuals with both migraine and prediabetes [183]. Besides specific treatment needed for diabetes, behavioral indications should be provided to headache patients for weight control, such as engaging in regular exercise and following a balanced daily dietary intake, considering the fact that diabetes often presents together with obesity [184], which in our review was found in 21 % (95 %CI: 17–26 %) of subjects. Topiramate, which has an appetite-suppressive effect and whose utilization has been associated to weight loss, may be considered as a prophylactic agent [185].

The association between headache disorders, most of all migraine, and Cardiovascular Diseases (CVDs) is well known since more than 40 years. A wide range of studies have revealed a link between migraine and hypertension, stroke, ischemic heart disease, patent foramen ovale and other cardiovascular diseases, as well as the role of migraine as a risk factors for several CVDs [36, 50]. Females aged 45 or less suffering from migraine with aura are exposed to an increased risk of stroke, particularly if smokers and if under oral contraceptives [186], and to an increased incidence of major CVD events, particularly if smokers and if they have comorbidities with hypertension and diabetes [35]. However, a relation between migraine and major CVD events has been shown also among men [117]. What is important to notice here, is that the association between migraine and stroke is stronger among young subjects than among older ones [77].

Evidence on the association between CVD and headache disorders other than migraine has been little reported. A recent study has shown that migraine sufferers undergoing pharmacologic treatment have a lower hazard of aneurysmal subarachnoid hemorrhage than subjects with TTH [141]. Our data, however, do not enable to address such a kind of relation as CVD comorbidities were addressed in their pooled prevalence to the entire group of headache sufferers.

The well-known and close relationship between migraine and CVD has many therapeutic implications. Among those for whom triptans are contraindicated, Gepants [187] as well as Lasmiditan, which showed a good safety profile in those with CVD [188], could be considered for acute migraine treatment. Migraine with aura should be considered as “red flag” risk factor for stroke, especially in young women who smoke and take oral contraceptives: thus, contraceptive therapies should be used with caution, if not avoided, among women suffering from migraine with aura, and specific advice for smoke cessation provided. Anyway, a thrombophilic assessment panel should be considered.

Prescription of prophylactic treatment should take into account the presence of cardiovascular comorbidities, leading to the exclusion of some preventive therapies (β-blockers in cardiac insufficiency, amitriptyline and calcium antagonists in arrhythmia, pizotifen in hypertension and angina), and some acute treatments (triptans in previous ischemic heart disease or hypertension, ergot-derivates in hypertension and vasculopathy). On the contrary, the use of β-blockers could be suggested as prevention therapy in headache sufferers with comorbidity to hypertension or angina, together with calcium antagonists and angiotensin inhibitors. Considering the new migraine-specific treatments, data emerging from trials with Calcitonin Gene-Related Peptide (CGRP) antibodies suggest that this specific blockade has shown no relevant cardiovascular side effects [189, 190]. Anti-CGRP and anti-CGRP receptor monoclonal antibodies, in addition to ditans and small molecule CGRP receptor antagonists (Second-Generation Gepants), have so far demonstrated efficacy and cardiovascular safety, further supporting the pathophysiological underpinnings of migraine as a primarily neuronal process.

The clinical vs. population subgroup analysis showed the existence of significant differences in comorbidities with high prevalence, such as anxiety and depressive disorders, which likely reflects different methodological approaches concerning the subject inclusion criteria, as well as the identification of such comorbidities. Participants enrolled in clinical studies might in fact undergo a clinical evaluation for such diseases or be stratified based on response to questionnaires for the evaluation of symptoms of depression or anxiety, such as the Major Depression Inventory or the Patient Health Questionnaire-9 [191, 192]. On the contrary, the identification of anxiety or depression cases in population studies, in addition to questionnaires’ use, likely relies on participants’ self-identification as depression or anxiety sufferers, a procedure that has a lower reliability [193]. Other differences favoring clinical studies deal with comorbidities which showed high prevalence only in clinical studies, such as fibromyalgia and RLS: in these cases, precise clinical criteria have to be applied, which makes it difficult to address them in population studies.

The sub-analyses carried out by age and gender group did not reveal unexpected findings. The association we produced here do not really reflect age and gender differences, but differences observed on the average age of subjects enrolled in the studies, above or below the age of 40.4, as well as on the prevalence of females in the single studies, above or below 77.8 %. In the case of age-based groups, the median age observed in the studies with younger participants was 36.9 (IQR: 33.7–38.8), and that in the studies with older participants was 46.8 (IQR: 42.2–52.8). In the case of gender-based groups, the median female percentage observed in the studies with less female participants was 71.2 % (IQR: 64.6–74.4 %), and that in the studies with more female participants was 89.6 % (IQR: 82.5–100 %). With these caveats in mind, it can be concluded that specific age and gender-based association can be found: hypertension is likely to be found as a comorbidity in studies whose participants are older and with higher men presence, whereas fibromyalgia, RLS, and depressive disorders are likely to be found as comorbidities in studies whose participants are younger and with higher female presence. These results are largely consistent with the available evidence that hypertension is more common in men and in older subjects, especially in high-income countries [194], and that depressive disorders are more common among younger females [195]. With regard to fibromyalgia and RLS, an association with female gender is known [196, 197], whereas the association with age is, on the contrary, debatable. Fibromyalgia is in fact more often diagnosed early in life and some evidence of rising prevalence with age exist [198]: however, among older subjects with chronic widespread pain, osteoarthritis rather than fibromyalgia is often diagnosed [196]. For RLS too an association with increasing age has been observed [197], which apparently contrasts with our analysis. A previous literature review addressing the comorbidity between migraine and RLS found significant differences between migraine and healthy controls with regard to RLS prevalence (17.6 % vs. 7.1 %): this suggested a specific pattern of association, which also include shared mechanisms of action involving the dopaminergic nucleus of the dorsalposterior hypothalamus [199]. In consideration of migraine epidemiology, which mostly affects younger females, and of the research design herein employed (i.e. the fact that we looked for comorbidities among headache disorders), our result showing an association with younger age in addition to female gender can be justified.

In order to address whether headache disorders are associated to a higher prevalence in the selected comorbidities, we contrasted such rates with the estimates generated by the GBD-2019 study. Such a choice was considered as the only viable since GBD estimates are referred to the global level, and we have global-level studies, and enable to produce age-standardized percentages [1]. In order to make the information referred to the estimates comparable to our results we would have to select, for each specific comorbidity, a different year of GBD estimates: in fact, our analysis span between 2000 and 2020, a period in which estimates for some conditions have significantly changed, and regional variation might be different. Moreover, publication year and data collection year are not identical. We therefore decided to rely on the last available ones. The comparison was made between the 95 %CI of our pooled prevalence and the 95 %UI of GBD-2019 estimates, with the latter being the results of a meta-analytic simulation performed in GBD studies.

Our results show that for some conditions, prevalence among headache sufferers was higher than in GBD-2019 estimates, which leads to concluding that headaches might be both a cause or a consequence of these comorbidities. For example, migraine can be a risk factor for several CVDs [36, 50]: thus it is not surprising that the pooled proportion of ischemic heart disease and of stroke/cerebrovascular conditions was higher among headache sufferers than among the general population. Another note can be made for comorbidities with conditions with a relevant pain component, such as back pain. Such an association has already been observed [200, 201] and might be due to the role of shared nociceptive ways, in particular dealing with the central sensitization, and cephalic and extracephalic allodynia [201].

The added value of the present literature review is that it enables a broader appreciation of the comorbidities of headache disorders. Most of available research, if not addressed in a meta-analytic way, point out few comorbidities or groups of conditions, and most of available knowledge was based on migraine only, with the result of excluding the recognition of a large set of comorbidities and of headache sufferers. The results herein presented reflect in part such a situation, and the reason for this is that the majority of the studies herein presented (100 out of 139) were on migraine only. However, we were able to produce new insights on minor conditions because we used the broadest possible approach, as we did not select a specific primary headache, we decided not to pre-select the main (and most known) comorbidities, and we included both clinical and population studies.

The comorbidities that have been identified in the present review are leading burdensome conditions in terms of disability-adjusted life years (DALYs) as shown in the last published estimates produced by the GBD study [1]. If all age groups are taken into account, ischemic heart disease ranked 2nd (accounting for 7.2 % of all-cause DALYs), stroke ranked 3rd (accounting for 5.7 % of all-cause DALYs), diabetes ranked 8th (accounting for 2.8 % of all-cause DALYs), and depressive disorders ranked 13th (accounting for 1.8 % of all-cause DALYs). If the 14–49 age group, where headache disorders are mostly prevalent, is instead taken into account, ischemic heart disease ranked again 2nd (accounting for 4.7 % of all-cause DALYs), depressive disorders ranked 6th (accounting for 3.5 % of all-cause DALYs), stroke ranked 9th (accounting for 3.2 % of all-cause DALYs), diabetes ranked 14th (accounting for 2.2 % of all-cause DALYs) and anxiety disorders ranked 15th (accounting for 2.0 % of all-cause DALYs). Therefore, addressing comorbidities of headache disorders with appropriate treatment, either pharmacological, behavioral or lifestyle-directed, may positively impact towards reducing the impact of some of the most burdensome diseases.

Some limitations have to be taken into account. First, we relied on Scopus only, rather than on a wider set of search engines, which might have caused a loss in studies’ identification. Second, we were unable to locate some studies, despite requests were sent to the corresponding authors. Third, we were unable to further address analyses by headache frequency, despite the relevance of comorbidities for the process of headache chronification: the reason for this is that only a minority of selected studies reported comorbidities by chronic vs. episodic headache or average headache frequency. Similarly, as very few studies presented comorbidity information on TTH and TACs, we were unable to present a comorbidity profile by primary headache. Fourth, around a fifth of studies included subjects with mixed populations, which makes it complex to understand the relation between headaches and comorbidities. Fifth, we used a taxonomy based on that employed by the GBD study consortium, which includes very broad labels for each comorbidity, with an unavoidable precision loss. Last, if on one side clinical studies have high reliability with the identification of specific headache disorders, on the basis of the second or third version of the International Classification of Headache Disorders, population studies are reasonably expected to be less precise.

## Conclusions

In conclusion, the results of this literature review with meta-analysis of comorbidities of primary headache disorders show that some of the most prevalent comorbidities of headache disorders – such as hypertension, back pain, anxiety and depression, diabetes, ischemic heart disease and stroke – are among the most burdensome conditions and relevant risk factors according to the GBD study together with headache disorders themselves. Many comorbidities could merely reflect coincidence of diseases that are common: however, the prevalence rate of some of them (e.g. back pain, sleep disorders, anxiety and depression) was higher when addressed as comorbidities of headaches compared to the general population estimates produced by GBD-2019. Therefore, addressing and treating the most relevant comorbidities of headache disorders not only positively impacts on the health status of headache sufferers, but it may also positively contribute towards reducing the impact of a group of high-burden conditions.

## Supplementary Information

**Additional file 1: Supplementary Table 1.** Search strategy. **Supplementary Table 2.** Distribution of comorbidities in all included studies and by main condition (raw mean and min-max percentage). **Supplementary figures, first set:** Sub-analysis by study type, clinical vs. population studies (the overall pooled proportion correspond to the overall proportion of headache sufferers with each specific comorbidity as described in table 2 of main text). **Supplementary figures, second set:** Sub-analysis by female proportion, < 77.8% vs. ≥ 77.8%. **Supplementary figures, third set:** Sub-analysis by average age, < 40.4 vs. ≥ 40.4 years.

## Data Availability

Not applicable.

## Abbreviations

CGRP: Calcitonin Gene-Related Peptide
CH: Cluster Headache
CM: Chronic Migraine
CVD: Cardiovascular Diseases
DALYs: Disability-Adjusted Life Years
GBD: Global Burden of Diseases, Injuries, and Risk Factors Study
IQR: Interquartile Range
PRISMA: Preferred Reporting Items for Systematic Reviews and Meta-Analyses
RLS: Restless Leg Syndrome
TTH: Tension-Type Headache
TACs: Trigeminal Autonomic Cephalalgias
YLDs: Years Lived with Disability
95%CI: 95% Confidence Intervals
95%UI: 95% Uncertainty Interval
