# Supplementary material for: Comorbidities of primary headache disorders: a literature review with meta-analysis
Source: J Headache Pain. 2021 Jul 14;22(1):71. doi: 10.1186/s10194-021-01281-z (PMC8278743; doi:10.1186/s10194-021-01281-z)
Supplement: Supplementary file 1 — Additional file 1: Supplementary Table 1. Search strategy. Supplementary Table 2. Distribution of comorbidities in all included studies and by main condition (raw mean and min-max percentage). Supplementary figures, first set: Sub-analysis by study type, clinical vs. population studies (the overall pooled proportion correspond to the overall proportion of headache sufferers with each specific comorbidity as described in table 2 of main text). Supplementary figures, second set: Sub-analysis by female proportion, < 77.8% vs. ≥ 77.8%. Supplementary figures, third set: Sub-analysis by average age, < 40.4 vs. ≥ 40.4 years. [file 10194_2021_1281_MOESM1_ESM.docx]

**Supplementary materials**

Caponnetto V, Deodato M, Robotti M, Koutsokera M, Pozzilli V, Galati C, Nocera G, De Matteis E, De Vanna G, Fellini E, Halili G, Martinelli D, Nalli G, Serratore S, Tramacere I, Martelletti P, Raggi A, On behalf of the European Headache Federation School of Advanced Studies (EHF-SAS). Comorbidities of primary headache disorders: a literature review with meta-analysis. J Headache Pain

**Supplementary Table 1.** Search strategy

| ( TITLE ( ( "Chronic Daily Headache" OR "Chronic Tension Type Headache" OR "Episodic Tension Type Headache" OR "Tension-Type Headache" OR tth OR headache OR "Headache Disorder*" OR "Medication Overuse Headache" OR moh OR migraine OR "Episodic Migraine" OR "Chronic Migraine" OR cm OR "Migraine Disorder" OR "Migraine With Aura" OR "Migraine Without Aura" OR "Transformed Migraine" OR "Cluster Headache" OR "Chronic Cluster Headache" OR "Episodic Cluster Headache" OR ch ) AND ( comorb* OR multimorb* OR alzheimer OR angina OR anx* OR arthritis OR arthrosis OR asthma OR atopic OR autism OR autoimmune OR bipolar OR "Brain injur*" OR "Brain Ischemia" OR "Brain tumor*" OR "Brain tumour*" OR "Brain cancer*" OR bronchitis OR cardiovascular OR celiac OR cerebrovascular OR "Chronic fatigue syndrome" OR conversion OR coronavirus OR covid OR depress* OR diabet* OR endometriosis OR epilepsy OR "Facial pain" OR fibromyalgia OR gastrointestinal OR "Head Injur*" OR hypertension OR hyperthyroidism OR hypothyroidism OR "Idiopathic Intracranial Hypertension" OR "Interstitial cystitis" OR "Irritable Bowel Syndrome" OR leukoaraiosis OR "Low Back Pain" OR meniere OR mental OR monogenic OR mood OR "Multiple Sclerosis" OR myocardial "Neck Pain" OR obesity OR "Orofacial Pain" OR "Painful bladder syndrome" OR panic OR parkinson OR "Post-traumatic stress disorder" OR ptsd OR raynaud OR "Restless Leg Syndrome" OR sleep OR somatoform OR stroke OR "Systemic lupus erythematosus" OR temporomandibular OR thyroid OR "Trigeminal neuralgia" OR vertigo OR vestibular ) ) ) OR ( ABS ( ( "Chronic Daily Headache" OR "Chronic Tension Type Headache" OR "Episodic Tension Type Headache" OR "Tension-Type Headache" OR tth OR headache OR "Headache Disorder*" OR "Medication Overuse Headache" OR moh OR migraine OR "Episodic Migraine" OR "Chronic Migraine" OR cm OR "Migraine Disorder" OR "Migraine With Aura" OR "Migraine Without Aura" OR "Transformed Migraine" OR "Cluster Headache" OR "Chronic Cluster Headache" OR "Episodic Cluster Headache" OR ch ) AND ( comorb* OR multimorb* OR alzheimer OR angina OR anx* OR arthritis OR arthrosis OR asthma OR atopic OR autism OR autoimmune OR bipolar OR "Brain injur*" OR "Brain Ischemia" OR "Brain tumor*" OR "Brain tumour*" OR "Brain cancer*" OR bronchitis OR cardiovascular OR celiac OR cerebrovascular OR "Chronic fatigue syndrome" OR conversion OR coronavirus OR covid OR depress* OR diabet* OR endometriosis OR epilepsy OR "Facial pain" OR fibromyalgia OR gastrointestinal OR "Head Injur*" OR hypertension OR hyperthyroidism OR hypothyroidism OR "Idiopathic Intracranial Hypertension" OR "Interstitial cystitis" OR "Irritable Bowel Syndrome" OR leukoaraiosis OR "Low Back Pain" OR meniere OR mental OR monogenic OR mood OR "Multiple Sclerosis" OR myocardial "Neck Pain" OR obesity OR "Orofacial Pain" OR "Painful bladder syndrome" OR panic OR parkinson OR "Post-traumatic stress disorder" OR ptsd OR raynaud OR "Restless Leg Syndrome" OR sleep OR somatoform OR stroke OR "Systemic lupus erythematosus" OR temporomandibular OR thyroid OR "Trigeminal neuralgia" OR vertigo OR vestibular ) ) ) AND ( LIMIT-TO ( SRCTYPE , "j" ) ) AND ( LIMIT-TO ( DOCTYPE , "ar" ) OR LIMIT-TO ( DOCTYPE , "sh" ) ) AND ( LIMIT-TO ( SUBJAREA , "MEDI" ) OR LIMIT-TO ( SUBJAREA , "NEUR" ) OR LIMIT-TO ( SUBJAREA , "NURS" ) OR LIMIT-TO ( SUBJAREA , "PHAR" ) OR LIMIT-TO ( SUBJAREA , "HEAL" ) OR LIMIT-TO ( SUBJAREA , "PSYC" ) OR LIMIT-TO ( SUBJAREA , "IMMU" ) OR LIMIT-TO ( SUBJAREA , "DENT" ) OR LIMIT-TO ( SUBJAREA , "MULT" ) OR LIMIT-TO ( SUBJAREA , "SOCI" ) ) AND ( EXCLUDE ( PUBYEAR , 1999 ) OR EXCLUDE ( PUBYEAR , 1998 ) OR EXCLUDE ( PUBYEAR , 1997 ) OR EXCLUDE ( PUBYEAR , 1996 ) OR EXCLUDE ( PUBYEAR , 1995 ) OR EXCLUDE ( PUBYEAR , 1994 ) OR EXCLUDE ( PUBYEAR , 1993 ) OR EXCLUDE ( PUBYEAR , 1992 ) OR EXCLUDE ( PUBYEAR , 1991 ) OR EXCLUDE ( PUBYEAR , 1990 ) OR EXCLUDE ( PUBYEAR , 1989 ) OR EXCLUDE ( PUBYEAR , 1988 ) OR EXCLUDE ( PUBYEAR , 1987 ) OR EXCLUDE ( PUBYEAR , 1986 ) OR EXCLUDE ( PUBYEAR , 1985 ) OR EXCLUDE ( PUBYEAR , 1984 ) OR EXCLUDE ( PUBYEAR , 1983 ) OR EXCLUDE ( PUBYEAR , 1982 ) OR EXCLUDE ( PUBYEAR , 1981 ) OR EXCLUDE ( PUBYEAR , 1980 ) OR EXCLUDE ( PUBYEAR , 1979 ) OR EXCLUDE ( PUBYEAR , 1978 ) OR EXCLUDE ( PUBYEAR , 1977 ) OR EXCLUDE ( PUBYEAR , 1976 ) OR EXCLUDE ( PUBYEAR , 1975 ) OR EXCLUDE ( PUBYEAR , 1974 ) OR EXCLUDE ( PUBYEAR , 1973 ) OR EXCLUDE ( PUBYEAR , 1972 ) OR EXCLUDE ( PUBYEAR , 1971 ) OR EXCLUDE ( PUBYEAR , 1970 ) OR EXCLUDE ( PUBYEAR , 1969 ) OR EXCLUDE ( PUBYEAR , 1968 ) OR EXCLUDE ( PUBYEAR , 1967 ) OR EXCLUDE ( PUBYEAR , 1966 ) OR EXCLUDE ( PUBYEAR , 1965 ) OR EXCLUDE ( PUBYEAR , 1964 ) OR EXCLUDE ( PUBYEAR , 1963 ) OR EXCLUDE ( PUBYEAR , 1962 ) OR EXCLUDE ( PUBYEAR , 1960 ) OR EXCLUDE ( PUBYEAR , 1959 ) OR EXCLUDE ( PUBYEAR , 1958 ) OR EXCLUDE ( PUBYEAR , 1957 ) OR EXCLUDE ( PUBYEAR , 1956 ) OR EXCLUDE ( PUBYEAR , 1954 ) OR EXCLUDE ( PUBYEAR , 1953 ) OR EXCLUDE ( PUBYEAR , 1951 ) OR EXCLUDE ( PUBYEAR , 1947 ) OR EXCLUDE ( PUBYEAR , 1946 ) OR EXCLUDE ( PUBYEAR , 1944 ) OR EXCLUDE ( PUBYEAR , 1941 ) OR EXCLUDE ( PUBYEAR , 1938 ) OR EXCLUDE ( PUBYEAR , 1934 ) OR EXCLUDE ( PUBYEAR , 1932 ) OR EXCLUDE ( PUBYEAR , 1930 ) OR EXCLUDE ( PUBYEAR , 1929 ) OR EXCLUDE ( PUBYEAR , 1928 ) OR EXCLUDE ( PUBYEAR , 1922 ) OR EXCLUDE ( PUBYEAR , 1914 ) OR EXCLUDE ( PUBYEAR , 1910 ) OR EXCLUDE ( PUBYEAR , 2000 ) ) AND ( EXCLUDE ( LANGUAGE , "Spanish" ) OR EXCLUDE ( LANGUAGE , "German" ) OR EXCLUDE ( LANGUAGE , "Chinese" ) OR EXCLUDE ( LANGUAGE , "French" ) OR EXCLUDE ( LANGUAGE , "Japanese" ) OR EXCLUDE ( LANGUAGE , "Russian" ) OR EXCLUDE ( LANGUAGE , "Polish" ) OR EXCLUDE ( LANGUAGE , "Italian" ) OR EXCLUDE ( LANGUAGE , "Portuguese" ) OR EXCLUDE ( LANGUAGE , "Turkish" ) OR EXCLUDE ( LANGUAGE , "Czech" ) OR EXCLUDE ( LANGUAGE , "Croatian" ) OR EXCLUDE ( LANGUAGE , "Bulgarian" ) OR EXCLUDE ( LANGUAGE , "Hungarian" ) OR EXCLUDE ( LANGUAGE , "Dutch" ) OR EXCLUDE ( LANGUAGE , "Persian" ) OR EXCLUDE ( LANGUAGE , "Korean" ) OR EXCLUDE ( LANGUAGE , "Serbian" ) OR EXCLUDE ( LANGUAGE , "Slovak" ) OR EXCLUDE ( LANGUAGE , "Danish" ) OR EXCLUDE ( LANGUAGE , "Hebrew" ) OR EXCLUDE ( LANGUAGE , "Norwegian" ) OR EXCLUDE ( LANGUAGE , "Romanian" ) OR EXCLUDE ( LANGUAGE , "Moldavian" ) OR EXCLUDE ( LANGUAGE , "Moldovan" ) OR EXCLUDE ( LANGUAGE , "Bosnian" ) OR EXCLUDE ( LANGUAGE , "Greek" ) OR EXCLUDE ( LANGUAGE , "Lithuanian" ) OR EXCLUDE ( LANGUAGE , "Swedish" ) OR EXCLUDE ( LANGUAGE , "Arabic" ) OR EXCLUDE ( LANGUAGE , "Ukrainian" ) OR EXCLUDE ( LANGUAGE , "Slovenian" ) OR EXCLUDE ( LANGUAGE , "Azerbaijani" ) OR EXCLUDE ( LANGUAGE , "Basque" ) OR EXCLUDE ( LANGUAGE , "Estonian" ) OR EXCLUDE ( LANGUAGE , "Finnish" ) OR EXCLUDE ( LANGUAGE , "Icelandic" ) OR EXCLUDE ( LANGUAGE , "Latvian" ) OR EXCLUDE ( LANGUAGE , "Thai" ) OR EXCLUDE ( LANGUAGE , "catalan" ) OR EXCLUDE ( LANGUAGE , "Undefined" ) ) AND ( EXCLUDE ( EXACTKEYWORD , "Adolescent" ) OR EXCLUDE ( EXACTKEYWORD , "Nonhuman" ) OR EXCLUDE ( EXACTKEYWORD , "Preschool Child" ) OR EXCLUDE ( EXACTKEYWORD , "Child, Preschool" ) OR EXCLUDE ( EXACTKEYWORD , "Infant" ) OR EXCLUDE ( EXACTKEYWORD , "School Child" ) OR EXCLUDE ( EXACTKEYWORD , "Animals" ) OR EXCLUDE ( EXACTKEYWORD , "Newborn" ) OR EXCLUDE ( EXACTKEYWORD , "Infant, Newborn" ) ) AND ( EXCLUDE ( EXACTKEYWORD , "Mortality" ) ) |
| --- |

**Supplementary Table 2.** Distribution of comorbidities in all included studies and by main condition (raw mean and min-max percentage)

| **Condition** |  | **All Included studies** | | |  | **By Condition** | |  |
| --- | --- | --- | --- | --- | --- | --- | --- | --- |
|  |  | **No. of studies** | **Median** | **Min-Max** |  | **Migraine only**  **[Median**  **(Min-Max)]** | **TTH, CH and other TACs, other primary headaches**  **[Median**  **(Min-Max)]** | |
| Depressive disorders |  | 51 | 36.9% | 11.7-99.3% |  | 38.2%  (12.2-99.3%) | 34.3%  (11.7-71.5%) | |
| Hypertension |  | 48 | 32.9% | 11.6-89.2% |  | 32.4%  (11.6-89.2%) | 35.7%  (14.3-84.3%) | |
| Anxiety disorders |  | 40 | 38.4% | 12.4-94.7% |  | 33%  (12.4-77.3%) | 48.4%  (16.7-94.7%) | |
| Diabetes mellitus |  | 39 | 32.4% | 11.5-89.2% |  | 29.2%  (11.5-86.8%) | 47%  (12.8-89.2%) | |
| Sleep disorder |  | 30 | 51.1% | 10-86.5% |  | 52.7%  (10-85.9%) | 47.9%  (11.5-86.5%) | |
| Stroke/Cerebrovascular |  | 30 | 32.9% | 10-98.1% |  | 33.9%  (10-98.1%) | 28.1%  (14.3-51.9%) | |
| Obesity |  | 26 | 36.4% | 14-96% |  | 36.5%  (14-96%) | 35.5%  (16.6-73.2%) | |
| Other cardiovascular and circulatory diseases |  | 23 | 42% | 11.9-100% |  | 40.2%  (13.3-97.3%) | 50.4%  (11.9-100%) | |
| Ischemic heart disease |  | 19 | 48.6% | 13.2-98% |  | 50.1%  (13.2-98%) | 22.8%  (-) | |
| Other neurological disorders |  | 19 | 41.1% | 13-87.4% |  | 42.1%  (18.5-81.6%) | 38.2%  (13-87.4%) | |
| Other mental disorders |  | 17 | 37.2% | 12.8-92.4% |  | 35.5%  (14.9-89.3%) | 40.2%  (12.8-92.4%) | |
| Restless leg syndrome |  | 16 | 43.8% | 11.7-100% |  | 47.1%  (17.3-100%) | 38.4%  (11.7-77.6%) | |
| Hyperlipidemia |  | 14 | 39.7% | 12.7-93.5% |  | 38.4%  (15.5-89.6%) | 44.2%  (12.7-93.5%) | |
| Other Metabolic/Kidney disease |  | 11 | 29.4% | 13.1-77.8% |  | 31.2%  (14.1-77.8%) | 21.6%  (13.1-30%) | |
| Any substance use disorder |  | 10 | 56% | 12.6-97.6% |  | 53%  (12.6-97.6%) | 68.3%  (59.6-76.9%) | |
| Oral disorders |  | 9 | 67.5% | 13.9-100% |  | 54.9%  (13.9-73%) | 92.6%  (77.8-100%) | |
| Thyroid diseases |  | 9 | 31.7% | 15.5-71.4% |  | 38.2%  (15.5-71.4%) | 23.6%  (16.7-29.7%) | |
| Fibromyalgia |  | 9 | 30.4% | 11.4-53.2% |  | 31.6%  (11.4-53.2) | 26.2%  (19.1-33.3%) | |
| Hypercholesterolemia |  | 8 | 50.7% | 11.7-99.9% |  | 41%  (11.7-78.9%) | 79.7%  (59.5-99.9%) | |
| Arthritis |  | 8 | 32.2% | 11.3-86.2% |  | 33.8%  (11.3-86.2%) | 27.4%  (21.6-33.3%) | |
| Allergies |  | 8 | 31.5% | 12.6-61.6% |  | 34.3%  (15.6-61.6%) | 12.6  (-) | |
| Asthma |  | 8 | 29.5% | 13.9-78.7% |  | 29.5%  (13.9-78.7%) | NA | |
| Back pain |  | 7 | 51.7% | 18.9-79.7% |  | 52%  (18.9-79.7%) | 50%  (-) | |
| Other digestive diseases |  | 7 | 35.4% | 11.5-85.4% |  | 43.7%  (15.5-85.4%) | 14.6%  (11.5-17.8%) | |
| Idiopathic epilepsy |  | 7 | 20.3% | 12.6-39.8% |  | 20.3%  (12.6-39.8%) | NA | |
| Post-Traumatic Stress Disorder |  | 7 | 20.1% | 11.3-26.7% |  | 22%  (17.9-24.8%) | 18.6%  (11.3-26.7%) | |
| Atrial fibrillation and flutter |  | 6 | 62.8% | 26.6-94.8% |  | 62.8%  (26.6-94.8%) | NA | |
| Other chronic respiratory diseases |  | 6 | 44.4% | 12.9-78.6% |  | 60.9%  (51.5-78.6%) | 27.9%  (12.9-37.4%) | |
| Any sense organ disease |  | 6 | 41.9% | 25-68.4% |  | 41.9%  (25-68.4%) | NA | |
| Bipolar disorder |  | 6 | 29.2% | 14.4-42.4% |  | 30%  (14.4-42.4%) | 27.4%  (16.3-38.5%) | |
| Parkinson disease |  | 5 | 36.5% | 11.3-94.8% |  | 65.8%  (36.9-94.8%) | 16.9%  (11.3-26%) | |
| Chronic obstructive pulmonary disease |  | 5 | 24.9% | 12.7-31.6% |  | 24.9%  (12.7-31.6%) | NA | |
| Chronic kidney disease |  | 5 | 22.1% | 12.3-47.4% |  | 28.1%  (13.8-47.4%) | 13%  (12.3-13.8%) | |
| Any skin and subcutaneous disease |  | 4 | 65.1% | 51.5-85.6% |  | 65.1%  (51.5-85.6%) | NA | |
| Other musculoskeletal disorders |  | 4 | 54.4% | 12.2-97.3% |  | 50.2%  (12.2-97.3%) | 68.8%  (-) | |
| Any other NCDs (Congenital birth defects & Gynecological diseases) |  | 4 | 36.5% | 14.7-60% |  | 36.5%  (14.7-60%) | NA | |
| Upper digestive system diseases |  | 4 | 34.3% | 17.1-61.2% |  | 39.2%  (17.1-61.2%) | 19.7%  (-) | |
| Irritable Bowel Syndrome |  | 4 | 29.9% | 11.5-53.8% |  | 28.2%  (11.5-53.8%) | 34.7%  (-) | |
| Hemoglobinopathies and hemolytic anemias |  | 4 | 24.1% | 15.5-34.7% |  | 25.1%  (15.5-34.7%) | 23.2%  (16.7-29.7%) | |
| Any cancer |  | 4 | 23.4% | 11.4-41.2% |  | 25.6%  (11.4-41.2%) | 16.7%  (-) | |

# **Supplementary figures, first set: Sub-analysis by study type, clinical vs. population studies (the overall pooled proportion correspond to the overall proportion of headache sufferers with each specific comorbidity as described in table 2 of main text).**

## Any cancer

## Ischemic heart disease

## Stroke/Cerebrovascular

## Hypertension

## Atrial fibrillation and flutter

## Other cardiovascular and circulatory disease

## Upper digestive system diseases

## Irritable bowel syndrome

## Other digestive diseases

## Diabetes mellitus

## Chronic kidney disease

## Obesity

## Hyperlipidemia

## Hypercholesterolemia

## Tyroid diseases

## Other metabolic/kidney disaese

## Arthritis

## Back pain

## Fibromyalgia

## Other musculoskeletal disorders

## Parkinson disease

## Idiopathic epilepsy

## Sleep disorder

## Restless leg syndrome

## Other neurological disorders

## Depressive disorders

## Bipolar disorder

## Anxiety disorders

## Post-traumatic stress disorder

## Other mental disorders

## Asthma

## Chronic obstructive pulmonary disease

## Other chronic respiratory diseases

## Any sense/organ diseases

## Any skin and subcutaneous disease

## Any substance use disorder

## Oral disorders

## Hemoglobinopathies and hemolytic anemias

## Other disorders (Congenital birth defects & Gynecological diseases)

## Allergies

# **Supplementary figures, second set: Sub-analysis by female proportion, <77.8% vs. ≥ 77.8%**

## Any cancer

## Ischemic heart disease

## Stroke/Cerebrovascular

## Hypertension

## Atrial fibrillation and flutter

## Other cardiovascular and circulatory disease

## Upper digestive system diseases

## Irritable bowel syndrome

## Other digestive diseases

## Diabetes mellitus

## Chronic kidney disease

## Obesity

## Hyperlipidemia

## Hypercholesterolemia

## Tyroid diseases

## Other metabolic/kidney disaese

## Arthritis

## Back pain

## Fibromyalgia

## Other musculoskeletal disorders

## Parkinson disease

## Idiopathic epilepsy

## Sleep disorder

## Restless leg syndrome

## Other neurological disorders

## Depressive disorders

## Bipolar disorder

## Anxiety disorders

## Post-traumatic stress disorder

## Other mental disorders

## Asthma

## Chronic obstructive pulmonary disease

## Other chronic respiratory diseases

## Any sense/organ diseases

## Any skin and subcutaneous disease

## Any substance use disorder

## Oral disorders

## Hemoglobinopathies and hemolytic anemias

## Other disorders (Congenital birth defects & Gynecological diseases)

## Allergies

# **Supplementary figures, third set: Sub-analysis by average age, <40.4 vs. ≥ 40.4 years.**

## Any cancer

## Ischemic heart disease

## Stroke/Cerebrovascular

## Hypertension

## Atrial fibrillation and flutter

## Other cardiovascular and circulatory disease

## Upper digestive system diseases

## Irritable bowel syndrome

## Other digestive diseases

## Diabetes mellitus

## Chronic kidney disease

## Obesity

## Hyperlipidemia

## Hypercholesterolemia

## Tyroid diseases

## Other metabolic/kidney disaese

## Arthritis

## Back pain

## Fibromyalgia

## Other musculoskeletal disorders

## Parkinson disease

## Idiopathic epilepsy

## Sleep disorder

## Restless leg syndrome

## Other neurological disorders

## Depressive disorders

## Bipolar disorder

## Anxiety disorders

## Post-traumatic stress disorder

## Other mental disorders

## Asthma

## Chronic obstructive pulmonary disease

## Other chronic respiratory diseases

## Any sense/organ diseases

## Any skin and subcutaneous disease

## Any substance use disorder

## Oral disorders

## Hemoglobinopathies and hemolytic anemias

## Other disorders (Congenital birth defects & Gynecological diseases)

## Allergies
